# Modulation of de Novo Lipogenesis Improves Response to Enzalutamide Treatment in Prostate Cancer

**DOI:** 10.3390/cancers12113339

**Published:** 2020-11-11

**Authors:** Mohamed Amine Lounis, Benjamin Péant, Kim Leclerc-Desaulniers, Dwaipayan Ganguli, Caroline Daneault, Matthieu Ruiz, Amina Zoubeidi, Anne-Marie Mes-Masson, Fred Saad

**Affiliations:** 1 Institut du Cancer de Montréal, Montréal, QC H2X 0A9, Canada; amine.lounis@umontreal.ca (M.A.L.); benjamin.peant.chum@ssss.gouv.qc.ca (B.P.); kim.leclerc-desaulniers.chum@ssss.gouv.qc.ca (K.L.-D.); anne-marie.mes-masson@umontreal.ca (A.-M.M.-M.); 2 Centre de Recherche du Centre Hospitalier de l’Université de Montréal (CRCHUM), Montreal, QC H2X 0A9, Canada; 3 Vancouver Prostate Centre, Vancouver, BC V6H 3Z6, Canada; dganguli@prostatecentre.com (D.G.); azoubeidi@prostatecentre.com (A.Z.); 4 Department of Urologic Sciences, Faculty of Medicine, University of British Columbia, Vancouver, BC V6T 1Z4, Canada; 5 Institut de Cardiologie de Montréal, Montreal, QC H1T 1C8, Canada; caroline_daneault@hotmail.com (C.D.); Matthieu.Ruiz@mhi-rc.org (M.R.); 6 Département de Nutrition, Université de Montréal (UdeM), Montreal, QC H3C 3J7, Canada; 7 Département de Médecine, Université de Montréal (UdeM), Montreal, QC H3C 3J7, Canada; 8 Département de Chirurgie, Université de Montréal (UdeM), Montreal, QC H3C 3J7, Canada

**Keywords:** prostate cancer therapy, combination therapy, de novo lipogenesis, lipid desaturation, cellular stress

## Abstract

**Simple Summary:**

Prostate cancer cells produce lipids via the activation of a specific pathway called fatty acid synthesis, also known as De novo lipogenesis. This pathway is essential for the survival and growth of most types of cancer cells, including prostate cancer. In our study, we showed that prostate cancer cells activate this lipid synthesis pathway to become more aggressive and develop resistance to commonly used therapeutic agents for advanced prostate cancer such as enzalutamide, an effective and commonly used androgen receptor (AR) targeted agent. Interestingly, by combining enzalutamide with a lipid synthesis pathway inhibitor, we were able to show that growth of prostate cancer tumors was more effectively reduced than with either agent alone. We also showed that this combination led to cell stress and death by changing the lipid content in the cell. These important findings could lead to new therapeutic strategies combining effective AR targeted therapies with lipid synthesis inhibitors for the treatment of advanced prostate cancer.

**Abstract:**

De novo lipogenesis (DNL) is now considered as a hallmark of cancer. The overexpression of key enzymes of DNL is characteristic of both primary and advanced disease and may play an important role in resistance to therapies. Here, we showed that DNL is highly enhanced in castrate resistant prostate cancer (CRPC) cells compared to hormone sensitive and enzalutamide resistant cells. This observation suggests that this pathway plays an important role in the initiation of aggressive prostate cancer and in the development of enzalutamide resistance. Importantly, here we show that both prostate cancer cells sensitive and resistant to enzalutamide are dependent on DNL to proliferate. We next combined enzalutamide with an inhibitor of Stearoyl CoA Desaturase 1 (SCD1), an important enzyme in DNL, and observed significantly reduced tumor growth caused by the important change in tumoral lipid desaturation. Our findings suggest that the equilibrium between monounsaturated fatty acids and saturated fatty acids is essential in the establishment of the more aggressive prostate cancer phenotype and that the combination therapy induces a disruption of this equilibrium leading to an important decrease of cell proliferation. These findings provide new insights into the role of DNL in the progression of prostate cancer cells. The study also provides the rationale for the use of an inhibitor of SCD1 in combination with enzalutamide to improve response, delay enzalutamide resistance and improve disease free progression.

## 1. Introduction

Prostate cancer (PC) is one of the most commonly diagnosed cancers and one of the leading causes of cancer death in men in the world [1,2]. Active surveillance, surgery and radiation are the main options for men diagnosed with localized PC. However, for men with advanced PC, the first line of treatment is most often androgen deprivation therapy (ADT) [3] involving surgical or medical castration. Most patients respond initially to ADT, but around 30% of patients relapse within 18–24 months [4]. This aggressive phase of the disease is referred to as castrate-resistant prostate cancer (CRPC) [5], which often maintains functional androgen receptor (AR) signaling that promotes PC by developing adaptive mechanisms including AR amplification, AR mutations, and expression of various AR splice variants [6,7]. Recent studies suggest that estrogen receptors (ERα and ERβ) may play an important role in PC progression and aggressiveness [8,9]. ERs appear to activate pathways implicated in the epithelial mesenchymal transition and may be involved in PC drug resistance [8,9]. Enzalutamide (ENZ) (Xtandi^®^, Astellas Pharma Canada, Markham, ON, Canada) effectively targets AR ligand binding domain, blocking its translocation to the nucleus and subsequently the AR binding to DNA [10,11]. Unfortunately, development of resistance to ENZ is also common and the molecular mechanisms that are involved remain unclear [12].

Cancer cells exhibit a high demand for metabolites such as glucose and lipids. In particular, lipids play an important role as a source of energy but also contribute to membrane building and act as secondary messengers for many molecular pathways [13]. To meet the greater demands for lipids, PC cells acquire the ability to activate de novo lipogenesis (DNL). This pathway is now considered a hallmark of cancer, and the overexpression of key DNL enzymes is characteristic of both primary and advanced disease [13]. DNL involves endogenous synthesis of triglycerides from acetyl CoA provided by glycolysis, encompassing multiple enzymatic steps. Briefly, carboxylation of acetyl CoA by acetyl CoA carboxylase (ACC) forms malonyl CoA, which is then converted by fatty acid synthase (FASN) into the primary fatty acid product: palmitate (C16:0). ELOVL6 elongates palmitate into stearate (C18:0), and both are converted into palmitoleate (C16:1) and oleate (C18:1) by stearoyl CoA desaturase 1 (SCD1), the enzyme that controls the rate-limiting step in the synthesis of monounsaturated fatty acids (MUFA). Palmitoleate and oleate are preferentially esterified into triglycerides for long term energy storage in lipid droplets, or into phospholipids for membrane formation [14].

AR signaling in PC regulates the expression of genes involved in proliferation and metabolic pathways, including DNL. Androgens activate lipid metabolism in PC through the induction of the SREBP transcription factor, the master positive regulator of the lipogenic genes FASN, ACC and SCD1 [15,16]. Alternatively, androgens can also activate lipid catabolism as confirmed by previous studies showing a high level of palmitate oxidation in PC cell lines [4,17]. Several studies have observed that targeting DNL results in a decrease in tumor growth and PC cell proliferation [18,19,20] as well as a protection against chemotherapy [21]. Recently, Zadra et al. [19] showed that a novel irreversible FASN inhibitor (IPI-9119) is able to change the cancer metabolome, particularly metabolites related to lipogenesis, and induce PC cell apoptosis. More interestingly, this drug mediates inhibition of full-length AR and AR variant expression [19]. These findings suggest that targeting DNL could be a promising strategy for enhancing ENZ treatment and to potentially prevent disease progression on ADT that leads to castration resistance.

In the present study, we sought to determine the effects of combining ENZ with an inhibitor of SCD1 in vitro as well as in an in vivo mouse model in order to prevent ENZ resistance. We also investigated the molecular pathways implicated in the observed effects and the evolution of lipid contents in tumors treated with this therapeutic approach.

## 2. Results

### 2.1. Lipid Synthesis Is Increased as Prostate Cancer Progresses from Hormone Sensitive to Castrate Resistant to Enzalutamide Resistant State

**Figure 1:**
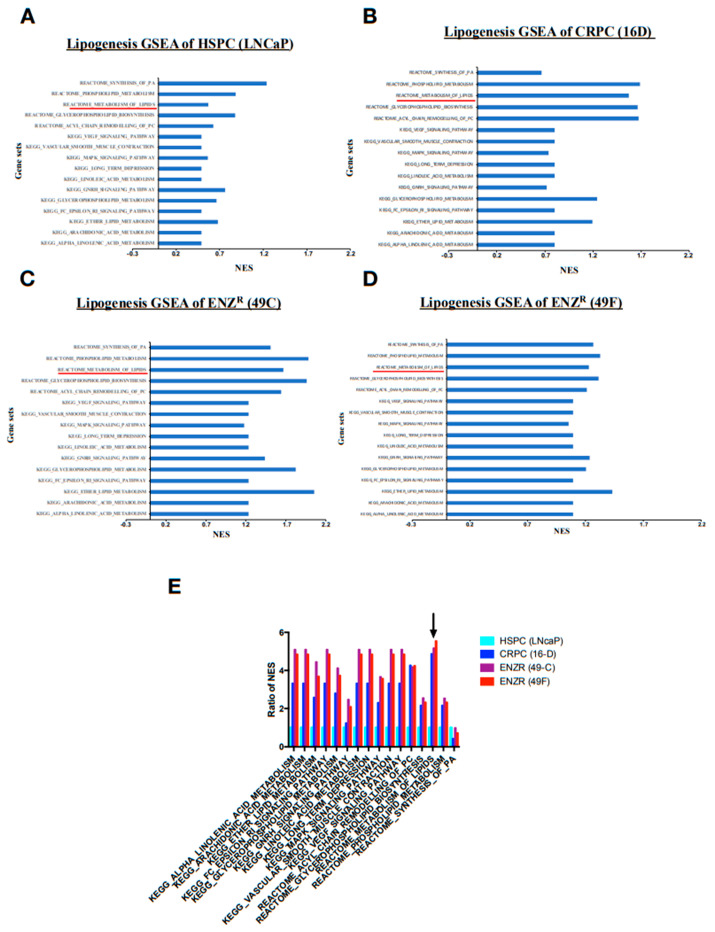
Lipid synthesis and particularly lipid metabolism are increased as prostate cancer evolves from HSPC to CRPC to ENZR CRPC. Graphs of gene set enrichment analysis of pathways implicated in lipid synthesis in HSPC cells (LNCaP) (**A**), CRPC cells (16D) (**B**), ENZR (49C) (**C**) and ENZR (49C) (**D**) (= 1). Fold change of NES (normalized enrichment score) of pathways implicated in lipid synthesis in CRPC cells and ENZR cells relative to HSPC cells. (**E**) The black arrow indicates change in genes related to lipid biosynthesis (Reactome Metabolism of lipids).

To understand changes in lipid metabolism associated with prostate cancer progression, RNAseq was performed in prostate cancer cell lines from hormone sensitive prostate cancer (HSPC) (LNCaP) to castrate resistant prostate cancer (CRPC) (16DCRPC) and enzalutamide resistant (ENZR) (49CENZR & 49FENZR). Gene set enrichment analysis was performed to identify biological pathways that were significantly altered. Interestingly, it was found that the majority of pathways implicated in the synthesis of lipids (lipogenesis) were enriched in the CRPC cell line (Figure 1A) as compared to the HSPC cell line (Figure 1B). In addition, based on normalized enrichment score, gene-sets related to lipogenesis were more enriched in ENZR cell lines (Figure 1C,D) as compared to the HSPC cell line. However, it was observed that genes related to lipid biosynthesis were marginally enriched in ENZR cell lines (Figure 1C,D) compared to CRPC cell lines (Figure 1B, black arrow).

### 2.2. De Novo Lipogenesis Is Increased in CRPC and ENZR Cell Lines and This Modulation Affects Survival of Prostate Cancer Cell Lines

**Figure 2:**
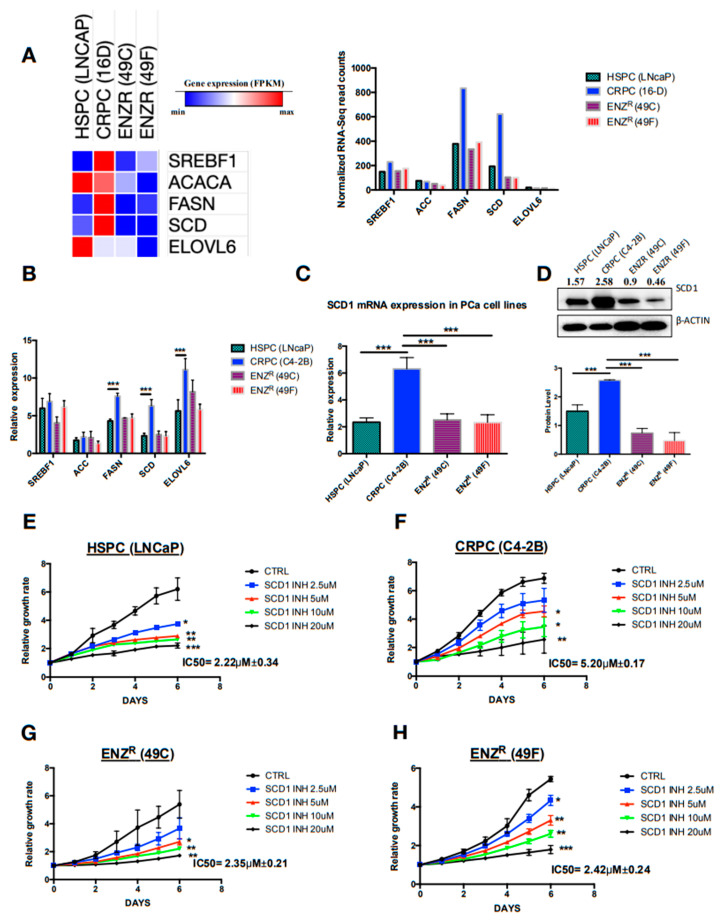
DNL is highly enhanced in CRPC and seems to be important in the PC aggressiveness and ENZ resistance. Heat map and graph showing RNA sequencing reads (RPKM) of genes implicated in DNL in HSPC cells (LNCaP), CRPC cells (16D) and ENZR cells (49C and 49F) (**A**); relative mRNA expression of genes implicated in DNL in HSPC cells (LNCaP), CRPC cells (C4-2B) and ENZR cells (49C and 49F) (**B**); relative mRNA expression (**C**) and protein expression (**D**) of SCD1 in HSPC cells (LNCaP), CRPC cells (16D) and ENZR cells (49C and 49F). Proliferation rates and percentage of cell proliferation of HSPC cells (LNCaP) (**E**), CRPC cells (C4-2B) (**F**), ENZR cells (49C) (**G**) and ENZR cells (49F) (**H**) were assessed using IncuCyte over six days of dose dependent treatment with the SCD1 inhibitor (SCD1 INH). The IC50 values of each cell line were calculated from dose–response curves using a GraphPad prism. Data represent the mean ± SD of three independent experiments. Data were analyzed using the one-way ANOVA test. * *p* < 0.05, ** *p* < 0.01, and *** *p* < 0.001.

A substantial enrichment of various pathways implicated in lipid synthesis in CRPC and ENZR cells as compared to HSPC was noted. Notably, we found that the metabolism of lipids was the pathway with the highest level of enrichment (Figure 1E). As DNL is an important pathway in lipid metabolism, a closer examination of differentially expressed genes of DNL using RNAseq analysis revealed a considerable increase in the expression of major genes in the CRPC and ENZR cell line compared to the HSPC cell line (Figure 2A). These data were further confirmer using qRT-PCR (Figure 2B). Importantly, the two genes highly expressed in CRPC cell lines compared to the HSPC and ENZR cell lines were FASN and SCD1 with more than a three-fold increase. We then focused on the mRNA and protein expression of SCD1 since it is known to play an important role in the synthesis of and equilibrium between saturated and monounsaturated fatty acids in mammalian cells [14]. The highest expression of SCD1 was confirmed in CRPC cell lines as compared to HSPC and ENZR cell lines (Figure 2C,D). More interestingly, after inhibition of the activity of SCD1 using a dose dependent specific inhibitor (A939572), it was shown that the HSPC cell line (LNCaP) (Figure 2E) and ENZR cell lines (49C and 49F) (Figure 2G,H) were very sensitive to the inhibition of SCD1 with “IC5Os of 2.22 μM ± 0.34, 2.35 μM ± 0.21 and 2.43 μM ± 0.24, respectively compared to CRPC cell line (C4-2B) IC5O of 5.20 μM ± 0.17” (Figure 2E). These data confirmed that SCD1 activity is important in PC survival and aggressiveness.

### 2.3. In Vitro Inhibition of SCD1 Combined with Enzalutamide Significantly Attenuates Prostate Cancer Cell Proliferation

**Figure 3:**
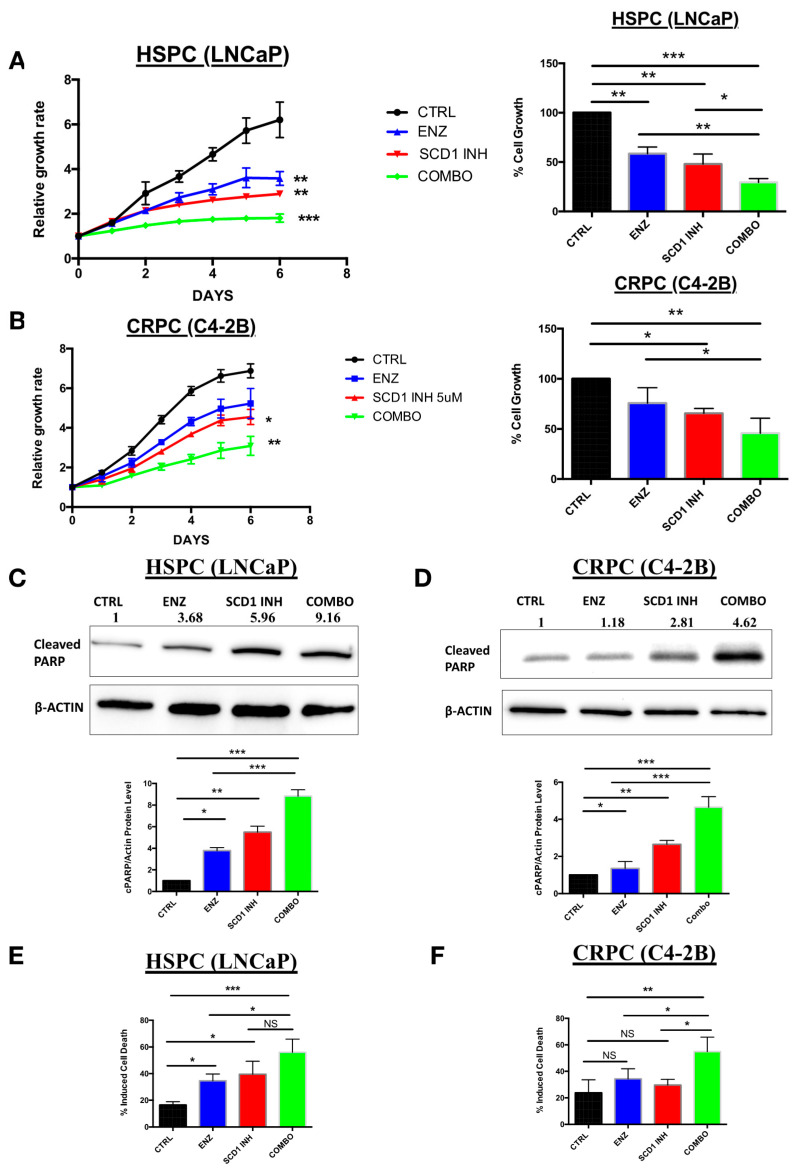
Inhibition of DNL combined with enzalutamide treatment has a greater impact on proliferation and induced higher cell death in prostate cancer cells than single-agent-based treatments. Proliferation rates and percentage of cell proliferation of PC cell lines LNCaP (**A**) and C4-2B (**B**) were assessed using IncuCyte over six days of treatment with either 10 μM enzalutamide (ENZ), 5 μM SCD1 inhibitor (SCD1 INH) or a combination of both (COMBO). Western blot analyses of cleaved PARP proteins from LNCaP (**C**) and C4-2B cells (**D**) treated for six days as previously described. Dead cell counts of LNCaP (**E**) and C4-2B cells (**F**) analyzed by flow cytometry after six days of treatment with 10 μM ENZ, 5 μM SCD1 INH or COMBO. Data represent the mean ± SD of three independent experiments. Data were analyzed using the one-way ANOVA test. * *p* < 0.05, ** *p* < 0.01 and *** *p* < 0.001.

Based on the observed increase in DNL in CRPC and ENZR cell lines, we hypothesized that targeting combining an inhibitor of lipogenesis (SCD1 inhibitor) with Enzalutamide may provide a good strategy in HSPC and CRPC. Our data showed a significant decrease in the proliferation of the HSPC cell line (LNCaP) treated with ENZ or SCD1 inhibitor alone (Figure 3A, blue and red curves) while combination therapy was far superior inducing and a drastic decrease in cell proliferation (Figure 3A, green curve). On the other hand, this effect was not as remarkable in CRPC cells (Figure 3B, blue and red curves) compared to cells treated with combination therapy (Figure 3B, green curve). Higher levels of cell death were also observed in HSPC (Figure 3C,E) and CRPC (Figure 3D,F) cell lines treated with COMBO compared to ENZ or SCD1 INH treatment alone. This observation was confirmed by an increase in the protein expression of cleaved PARP, a marker of apoptosis (Figure 3E,F).

To confirm that the observed effects were due to the specific inhibition of SCD1, transfections were performed using either scramble siRNA, siRNA SCD1 or pcDNA SCD1 plasmids and cells were treated with or without ENZ (Figure S1A). Inhibition of SCD1 by siRNA in the presence of ENZ induced a similar decrease in cell proliferation as observed with the COMBO using SCD1 INH. More importantly, overexpression of SCD1 using specific pcDNA-SCD1 (pcSCD1) plasmid rescued both HSPC cell line (LNCaP) (Figure S1B) and CRPC cell line (C4-2B) (Figure S1C) from inhibition of cell proliferation induced by ENZ.

### 2.4. Combination Therapy Induces Significant ER Stress and Produces High Levels of Reactive Oxygen Species Associated with PC Cell Death

**Figure 4:**
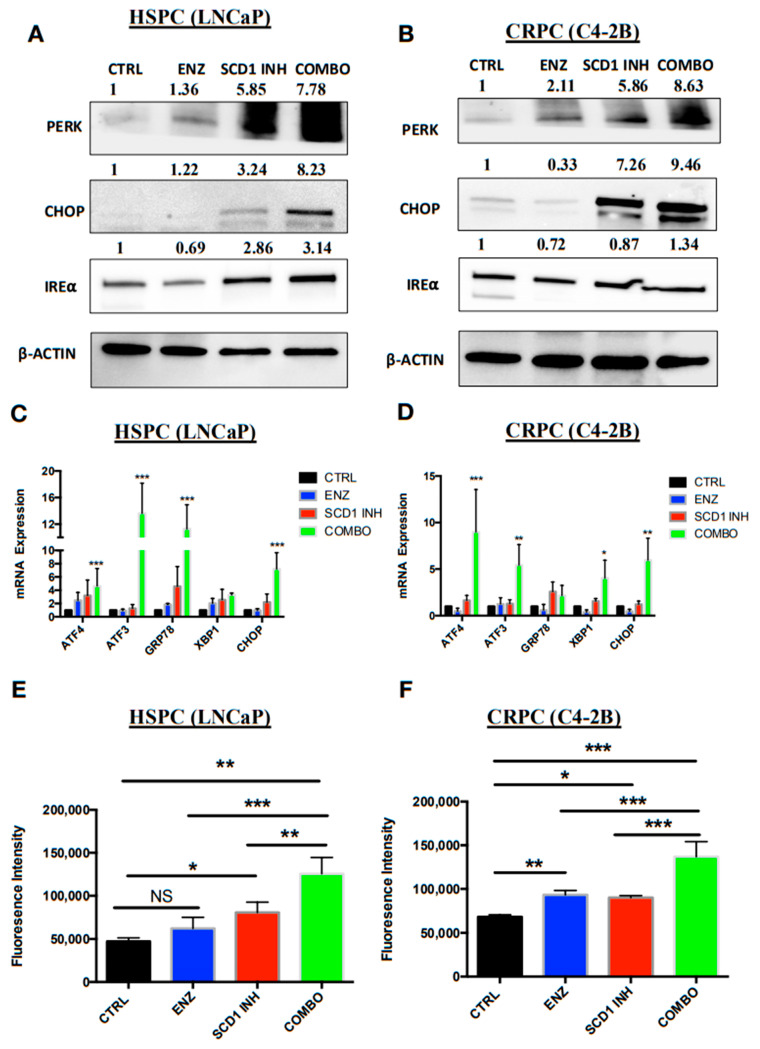
COMBO treatment induces high levels of ER stress and ROS associated with a more pronounced cell death phenotype. Expression of proteins (**A**,**B**) and mRNA (**C**,**D**) of ER stress markers was assessed in LNCaP and C4-2B cells untreated (CTRL) and treated with ENZ (10 μM), SCD1 INH (5 μM) or both (COMBO) for six days. Graphs represent levels of ROS production in treated LNCaP (**E**) and C4-2B cells (**F**). Data were analyzed using the one-way ANOVA test. * *p* < 0.05, ** *p* < 0.01 and *** *p* < 0.001.

Previous studies have shown that inhibition of DNL in various cells induces ER stress and high levels of ROS production [19,22]. To confirm this, protein and mRNA expression of key markers of the different steps of ER stress were analyzed and the level of ROS production quantified to identify a similar phenotype in our cell lines. The COMBO treatment induced an increase in expression levels of the RE stress proteins PERK, CHOP and IRE⍺ in the HSPC cell line (LNCaP) (Figure 4A). However, in the CRPC (C4-2B) cells treated with COMBO, a substantial increase in PERK and CHOP expression was observed and a slight increase in the IRE⍺ expression levels (Figure 4B) compared to cells treated with ENZ or SCD1 INH alone. The mRNA expression of ER stress markers ATF3, ATF4, GRP78 and CHOP increased in HSPC (LNCaP) cells treated with COMBO as compared to ENZ or SCD1 INH alone (Figure 4C). In addition, a notable increase in ATF3, ATF4, XBP1 and CHOP was observed in CRPC (C4-2B) cells treated with COMBO as compared to ENZ or SCD1 INH alone (Figure 4D). Finally, compared to cells treated with ENZ or SCD1 INH alone, COMBO treatment induced a high level of cellular ROS in both HSPC (LNCaP) and CRPC (C4-2B) cells (Figure 4E,F).

### 2.5. Combination Therapy Induces Inhibition of the Oncogenic P13K/AKT Pathway via a Decrease in Cellular Oleate

**Figure 5:**
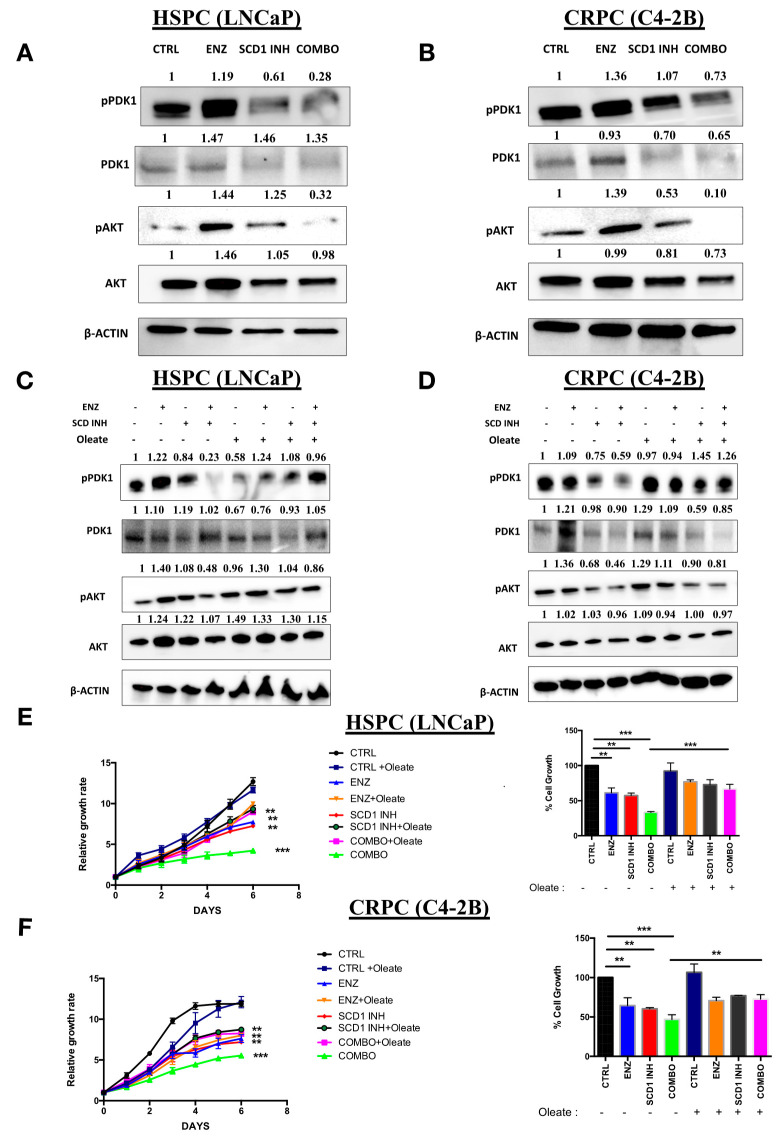
COMBO treatment affects the PI3K/AKT pathway in prostate cancer cell lines. Western blot analyses of phosphorylated PDK1 (pPDK1), PDK1, phosphorylated AKT (pAKT) and AKT proteins from LNCaP (**A**) and C4-2B (**B**) cells untreated (CTRL) and treated with 10 μM ENZ, 5 μM SCD1 INH or both (COMBO) for six days. Western blots of pAKT and AKT proteins of LNCaP (**C**) and C4-2B (**D**) treated with 10 μM ENZ, 5 μM SCD1 INH or COMBO in the presence or absence of 30 μM oleate for 6 days. The pAKT ratio represents pAKT/AKT and pPDK1 ratio represents pPDK1/PDK1. Proliferation of PC cells LNCaP (**E**) and C4-2B (**F**) was assessed using IncuCyte over six days of treatments with 10 μM ENZ, 5 μM SCD1 INH or COMBO in the absence or presence of 30 μM oleate (OL). Bar graphs show cell proliferation of treated LNCaP and C4-2B at day 6. Data represent the mean ± SD of three independent experiments. Data were analyzed using the one-way ANOVA test. ** *p* < 0.01 and *** *p* < 0.001.

The PI3K/AKT pathway has a major role in PC proliferation and in the regulation of various metabolic processes including DNL [23]. Surprisingly, an increase in the phosphorylation of AKT and PDK1 was observed in both the HSPC (LNCaP) (Figure 5A) and CRPC (C4-2B) (Figure 5B) cells treated with ENZ treatment alone. In contrast, SCD1 INH treatment induced a decrease in the phosphorylation of AKT and PDK1 in HSPC (LNCaP) (Figure 5A) and CRPC (C4-2B) cells (Figure 5B). Importantly, COMBO treatment led to a strong inhibition of both AKT and PDK1 in the two cell lines (Figure 5A,B). This suggests that PC cells in the presence of ENZ treatment are still able to adapt via a molecular compensation by increasing the PI3K/AKT pathway. Our study also suggested that MUFA may play an important role in the regulation of the AKT pathway. To confirm this hypothesis, cells were treated with the same treatments (CONTROL, ENZ, SCID1 INH, COMBO) in the absence or presence of oleate, a major product of SCD1. Interestingly, the addition of oleate to the COMBO treatment induced a rescue of the phosphorylation of AKT and PDK1 in HSPC (LNCaP) (Figure 5C) and CRPC (C4-2B) cells (Figure 5D) as compared to COMBO treatment alone. More importantly, the addition of oleate to the medium of cells treated with COMBO restored cell proliferation as compared to COMBO treatment alone (Figure 5E,F). These results suggest that oleate may play an important role in the activation of PI3K/AKT pathway and that cancer cells require a minimal amount of oleate to proliferate.

### 2.6. Pharmacological Combination of Enzalutamide and SCD1 Inhibitor Targets Tumor Growth More Efficiently than Monotherapy in Prostate Cancer Xenograft Models

**Figure 6:**
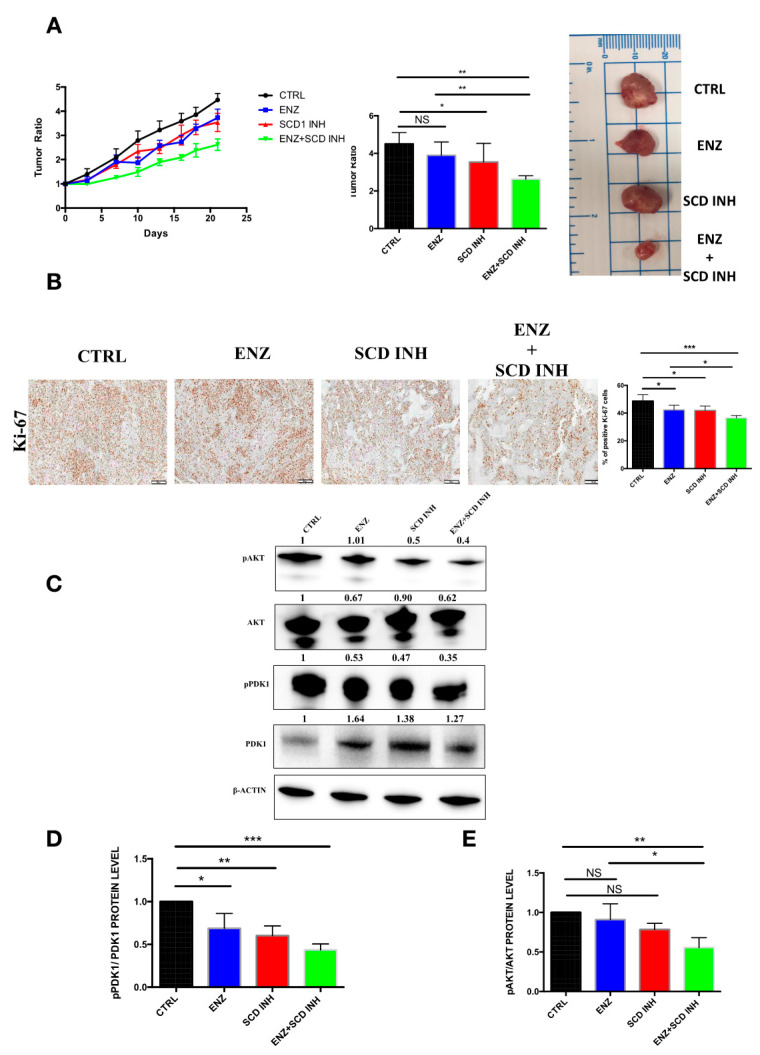
Combination of therapies suppresses tumorigenesis and inhibits the PI3K/AKT pathway in C4-2B prostate cancer xenografts tumors. (**A**) tumor ratio (growth rate: represents tumor volumes at different times divided by initial tumor volumes at first day before treatments) at different times during treatment for CTRL, ENZ (20 mg/kg), SCD1 INH (30 mg/kg) or the combination groups (left panel), tumor ratio at end of treatment (middle panel) and image of representative tumors removed from mice (*n* = 8–10 mice per group—right panel); (**B**) Ki67 expression and quantification in xenograft tissue of different treatment groups C4-2B (5 tumors per group randomly selected); (**C**–**E**) Western blot images (**C**) and expression levels of phosphorylated PDK1 (**D**) and phosphorylated AKT (**E**) in tumors after treatment (five tumors per group randomly selected). The pAKT ratio represents pAKT/AKT and pPDK1 ratio represents pPDK1/PDK1. NS: not significant. Data were analyzed using the one-way ANOVA test. * *p* < 0.05, ** *p* < 0.01 and *** *p* < 0.001.

To validate our cell culture observations in a preclinical setting, the effects of different treatments were investigated in mouse models of PC. Murine xenografts were derived from C4-2B cells. A significant decrease in tumor growth was observed in mice treated with the combination of therapies (ENZ+SCD1 INH) as compared to untreated mice (CTRL) or mice treated with ENZ or SCD1 INH alone (Figure 6A). As observed in the cell lines, tumors of mice treated with the combination showed a marked decrease in the expression of the cell proliferation marker Ki67 as compared to those treated with ENZ or SCD1 INH alone (Figure 6B). The combination of therapies resulted in a decrease of AKT and PDK1 phosphorylation in the xenografts of mice treated with this combination as compared to those treated with ENZ and SCD1 INH alone (Figure 6C–E). This decrease is likely one of the key elements involved in the inhibition of proliferation and tumor growth.

### 2.7. Combination Therapy Induces Changes in the Monounsaturated and Saturated Fatty Acid Content in Prostate Cancer Tumors

**Figure 7:**
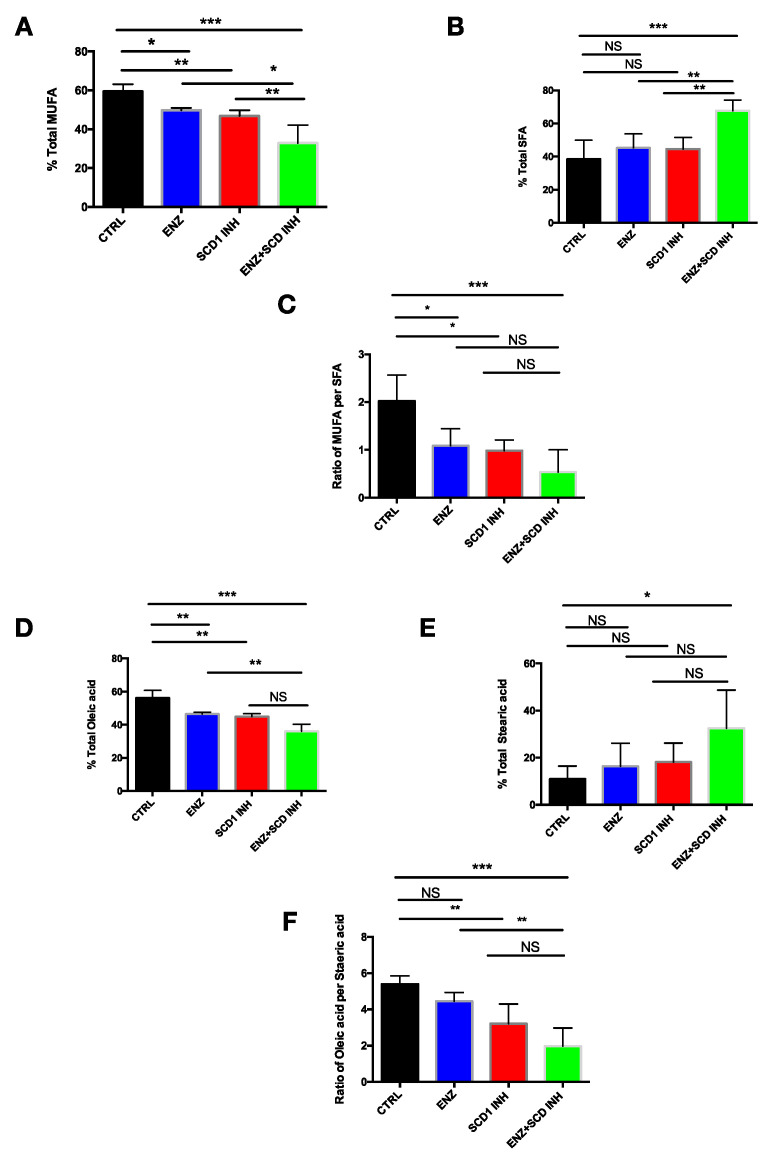
Combination of therapies induces changes in the monounsaturated and saturated fatty acids content in C4-2B xenograft models. (**A**) Percentage of total MUFA, (**B**) SFA and (**C**) ratio of MUFA/SFA in tumors after 21 days of treatment for CTRL, ENZ (20 mg/kg), SCD1 INH (30 mg/kg) or the combination groups; 3–5 tumors per group (randomly selected); (**D**) percentage of total oleic acid (C18:1), (**E**) stearic acid (C18:0) and (**F**) ratio of C18:1/C18:0 in C4-2B xenograft models after 21 days of treatment. NS: not significant. Data were analyzed using the one-way ANOVA test. * *p* < 0.05, ** *p* < 0.01, and *** *p* < 0.001.

To investigate the lipid profile changes in xenografts from mice untreated or treated with ENZ, SCD1 INH or with both, a lipidomic approach was used to quantify total fatty acids. As expected, ENZ induced a decrease in MUFA content, MUFA/saturated fatty acid (SFA) ratio and an increase in SFA (Figure 7A–C). The same results were observed in mice treated with the SCD1 INH. Importantly, a more substantial decrease of MUFA levels was observed in the tumors of mice treated with the combination of therapies as compared to ENZ or SCD1 INH alone (Figure 7A). However, a strong increase of SFA content (Figure 7B) and, consequently, a decrease in the MUFAs/SFA ratio was observed (Figure 7C).

Based on these results, the analysis of the different lipid fractions in the tumors of mice treated with combination therapy showed that the major fatty acids affected were oleic acid (C18:1) with a decrease of around 40% (Figure 7D) and stearic acid (C18:0) with an increase of more than 150% (Figure 7E). The ratio of C18:1/C18:0 was also significantly decreased in tumors treated with the combination therapy (Figure 7F). Together these results suggest that ENZ therapy in combination with SCD1 inhibition preferentially affects the desaturation of stearic acid leading to a marked decrease of oleate and an increase in stearic acid that may contribute to ER stress and a decrease in PC cell proliferation via the inhibition of the PI3K/AKT pathway.

## 3. Discussion

DNL is considered a hallmark of cancer pathogenesis [13,14]. Previous studies have shown that inhibition of key actors of DNL reduces PC growth and progression [18,19,20]. Our study is the first to show that lipid synthesis plays an important role in PC aggressiveness and progression. We showed that CRPC cells exhibit a significant increase in DNL as compared to HSPC and ENZR cells. Importantly, targeting DNL, via the inhibition of SCD1, considerably reduces PC growth suggesting an important role of this pathway in PC progression and survival. DNL appears to be important in the progression of PC to a more aggressive and resistant phenotype. To reverse the increase of DNL and prevent ENZ resistance, we tested the effect of the combination of SCD1 inhibitor and ENZ treatment in HSPC and CRPC cells. We demonstrated that this combination considerably inhibits the growth of PC xenografts. This inhibition of proliferation of PC was associated with reduced levels of cellular MUFA, in particular oleate, and increased levels of SFA, leading to an inhibition of the PI3K/AKT pathway, an induction of ER stress and high levels of ROS. This discovery sheds new light on the deregulation of the MUFA/SFA ratio in the activation of the oncogenic PI3K/AKT pathway and on the regulation of cellular stress in PC.

Enzalutamide (ENZ) is a novel efficient AR targeted therapy for advanced PC and has also been used recently as a first line therapy for hormone sensitive PC patients [24]. However, consistent with the majority of drugs used in PC, almost all patients will eventually develop resistance to Enzalutamide and new strategies are needed to prevent or circumvent this resistance. In our study, we showed that DNL may play an important role in PC progression and the development of resistance. We showed that the combination of SCD1 INH and ENZ is more effective than either agent alone and that proliferation decreased not only in androgen sensitive PC cell lines (LNCaP) but also in castrate-resistant cell lines (C4-2B). Several studies have reported that DNL is initially suppressed after castration, followed by a reactivation during the emergence of CRPC, suggesting that DNL is a key survival pathway that can promote resistance to ADT [25,26]. Interestingly, a recent study correlated DNL activation and the reactivation of AR signaling in cells resistant to Enzalutamide and in PC tumors from patients that relapsed following Enzalutamide treatment [26]. One potential mechanism for the reactivation of DNL genes is the emergence of constitutively active AR splice variants (AR-Vs), such as AR-V7. In our study, we showed that inhibition of SCD1 in combination with enzalutamide induces changes in the MUFA/SFA ratio. One of the potential consequences of this altered desaturation ratio is changes in membrane fluidity [27] leading to more efficient drug uptake. It has also been shown that AR is regulated by SREBP1, a major transcriptional regulator of DNL enzymes, via binding of SREBP1 upstream of the AR gene. This feedback loop activates the expression of DNL enzymes as well as AR expression and function [28]. Based on the decreased level of oleate in tumors (Figure 7), previous studies suggest that this results in the activation of SREBP1 [29], probably leading to a decrease in AR and ARV-7 transcription.

Consistent with these observations, another study showed a similar effect using a combination therapy using a newly synthetized inhibitor of FASN with Enzalutamide treatment in 2D and 3D PC cell lines [19]. The authors demonstrate that the inhibition of DNL using a FASN inhibitor results in a decrease in the transcription of both full-length AR and the splice variant AR-V7. However, FASN inhibition markedly affects the levels of various fatty acids in cells compared to SCD1 inhibition that only disrupts the equilibrium between saturated and monounsaturated fatty acids [30].

To our knowledge, our study is the first to confirm that the combination of ENZ and SCD1 INH can induce greater tumor regression of murine xenografts of PC cell lines compared to each treatment alone. This original preclinical study provides strong evidence that DNL may play an important role in recurrence/resistance and that inhibiting DNL in combination with commonly used AR-targeted therapies, such as enzalutamide, could be a promising first line approach in the management of PC. The effect of this therapy may involve the induction of more cellular ER stress and ROS production, and greater apoptosis than single-agent treatment. Modulation of lipid metabolism and membrane lipid composition as well as inhibition of SCD1 activity have all been reported to trigger ER stress [19,31,32], and previous studies have linked ER stress with ROS production [33,34]. Indeed, an increase of ER stress markers and a high level of unfolded protein response (UPR) in cells induces a higher production of ROS, which in turn maintains the increase in ER stress markers. An increase of both ER stress and/or ROS production has been correlated with an increase in apoptosis [22,35,36]. Together, these findings suggest that inhibition of SCD1 activity contributes to an increase in ER stress leading to a higher production of ROS, and that the sum of these effects leads to a much higher degree of apoptosis in cells treated with combination therapy than with monotherapy.

Finally, we found that the combination of ENZ and SCD1 INH reduced AKT phosphorylation levels which inhibited the activation of the PI3K/AKT pathway, an important oncogenic pathway that regulates cell growth, proliferation and apoptosis [37,38]. This pathway has also been shown to contribute to the development of drug resistance in PC and other cancers [39,40,41]. Inhibition of this pathway was reversed by the addition of oleate, a major product of SCD1. We also showed the same effect when we overexpressed SCD1 in cells treated with ENZ. These results suggest that this combination induces important changes in fatty acid content that may inactivate a major oncogenic pathway in PC. Specifically, we showed an important decrease in MUFA, particularly oleate, in tumors. Moreover, an increase in SFA was also observed in these tumors as compared to the untreated tumors or tumors treated with monotherapies. These findings confirm previous observations suggesting that oleate plays an important role in the regulation of the PI3K/AKT pathway [42,43,44]. Cellular oleate in cancer cells has been reported to activate the PI3K/AKT pathway and enhance tumor growth and cell migration of cancer cells. However, blocking the activity of SCD1 (an oleate producer) in various cancer cells induced the opposite effect characterized by a decrease in tumor proliferation, migration and an increase in apoptosis [20,45,46,47,48]. Our observations confirm the reports that oleate may play an important role in cancer pathogenesis and development. Furthermore, studies have shown that an increase in SFA content in cells induces an increase in ER stress and apoptosis [49,50,51,52], which supports our findings of increased SFA, ER stress and apoptosis induced by the combination of treatments. Hence, inhibiting SCD1 in combination with ENZ significantly changes the lipid content in PC cells and renders them more susceptible to cellular stress and apoptosis.

Mechanistically, we have demonstrated that the combination therapy affects the lipid content in tumor cells by causing a decrease in MUFA that affects the activation of the PI3K/AKT pathway, a major oncogenic pathway in PC, resulting in diminished cell proliferation. This effect is further compounded by an increase in SFA content inducing ER stress and a high level of ROS content, resulting in greater cell death.

## 4. Materials and Methods

### 4.1. Reagents

Enzalutamide (ENZ; Xtandi^®^, Astellas Pharma Canada, Markham, ON, Canada) and 4-(2-Chlorophenoxy)-*N*-(3-(methylcarbamoyl-phenyl)piperidine-1-carboxamide (A939572, Biofine International Inc., Vancouver, BC, Canada) were resuspended in 100% DMSO for cell culture use. For mouse studies, A939572 was solubilized in simple syrup 8% sucrose (Sweeting Vehicule, Laboratoire Atlas, Anjou, QC, Canada) and ENZ in 0.9% sodium chloride buffer containing 10% DMSO, 0.5% methyl cellulose, and 0.1% Tween 80.

### 4.2. Cell Culture and Transfection

HSPC cell line LNCaP and CRPC cell line C4-2B were purchased from American type culture collection (ATCC). CRPC cell line (16D) and CRPC ENZR Cell lines (49C and 49F) have previously been described [53]. Cells were cultured in RPMI-1640 (Wisent Bio products, Saint-Jean-Baptiste, QC, Canada) supplemented with 10% fetal bovine serum (FBS) (Wisent Bio products, Saint-Jean-Baptiste, QC), 100 U/mL penicillin-G, 100 mg/mL streptomycin (Wisent Bio products, Saint-Jean-Baptiste, Qc ) and incubated at 37 °C in a humidified atmosphere containing 5% CO2. For CRPC ENZR Cells, 10 μM of ENZ was added to the medium. Cells were treated with DMSO (CTRL), 10 μM ENZ, 5 μM of the SCD1 inhibitor (SCD1 INH) A939572 or a combination of both 10 μM ENZ and 5μM SCD1 INH (COMBO) for 6 days. In parallel, cells were transfected using either scramble siRNA (ctrl) or SCD1 siRNA (LQ-005061-00-0002, Dharmacon, Lafayette, CO, USA) for 3 days. We also transfected HSPC (LNCaP) and CRPC (C4-2B) cells with either pcDNA control or pcDNA SCD1 plasmids (generous gift from Dr. Mounier, Departement of Biological Sciences, University of Quebec At Montreal (UQAM)). All transfection procedures were performed using lipofectamine 2000 (Invitrogen, Carlsbad, CA, USA) according to the manufacturer’s protocol.

### 4.3. RNAseq

Total RNA was extracted using the PureLink RNA kit (ThermoFisher Scientific, Waltham, MA, USA) a library was constructed using NEBnext Ultra ii Stranded RNA Library Prep Kit (New England Biolabs Inc., Whitby, ON, Canada) and sequencing was performed on an NextSeq 500 (Illumina, San Diego, CA, USA) (42 × 42-bp paired-end reads).

### 4.4. Gene Set Enrichment Analysis (GSEA)

Software from the Broad Institute, (Cambridge, MA, USA) was used for pathway analysis to identify differentially expressed genes in the Molecular Signature database, version 7.1 run in classic mode. Pathways enriched with a nominal *p*-value < 0.05 and false discovery rate < 0.25 were considered to be significant. Single sample GSEA (ssGSEA) was carried out using the ssGSEAProjection.

### 4.5. IncuCyte Cell Proliferation Phase Contrast Imaging Assay

For proliferation of HSPC (LNCaP), CRPC (C4-2B) and CRPC ENZR (49C and 49F) cells, 2000 cells/well were seeded in 96-well plates. Cells were treated, or transfected as described above, and incubated for 6 days. Plates were imaged by phase contrast using the IncuCyteTM Live Cell Imaging System (Essen BioScience^®^, Ann Arbor, MI, USA). Frames were captured at 2 h-intervals for 6 days from two separate regions/well using a 10X objective. Proliferation growth curves were constructed using IncuCyteTM Zoom software (Essen BioScience^®^, Ann Arbor, MI, USA). Each experiment was performed in triplicate and repeated three times.

### 4.6. Protein Extraction and Western Blot Analysis

Cells were harvested in 1% Triton lysis buffer (150 mM NaCl, 20 mM Tris HCl, 1 mM EDTA, 1 mM EGTA, 1% Triton X-100, 1% deoxycholate, at pH 7.4). All lysis buffers were supplemented with 1% protease and phosphatase inhibitor cocktail (ThermoFisher Scientific, Waltham, MA, USA). Protein samples were resolved on SDS-polyacrylamide gel electrophoresis (SDS-PAGE) and transferred onto nitrocellulose membranes. Membranes were blocked with 5% non-fat dry milk resuspended in TBS buffer and probed with appropriate antibodies (Table S1). This was followed by incubation in 5% non-fat dry milk in TBS-Tween buffer with horseradish peroxidase (HRP)-conjugated secondary antibodies: anti-rabbit (#7074, Cell Signaling Technology Inc., Danvers, MA, USA) diluted 1:10,000 or anti-mouse (#7076, Cell Signaling, Danvers, MA, USA) diluted 1:10,000. All immunoblots were visualized using Amersham ECL Western blotting detection reagent (ThermoFisher Scientific, Waltham, MA, USA) and images taken using the ChemiDoc imaging system (Biorad, Saint-Laurent, QC, Canada). The whole blot can be found at Figure S2.

### 4.7. Oxidative Stress Detection (ROS)

In order to verify whether exposure of HSPC (LNCaP) and CRPC (C4-2B) cells differed in their response to different treatments leading to oxidative stress, intracellular reactive oxygen species (ROS) was first detected by the ROS assay kit with 2′,7′-dichlorodihydrofluorescein diacetate (ab113851, DCFDA Cellular ROS Detection Assay Kit, Abcam, Toronto, ON, Canada). Briefly, cells were seeded into 96-well plates in serum-free medium without phenol red. After 6 days of treatments with ENZ, SCD1 INH or both (COMBO) cells were incubated in the dark for 45 min at 37  °C with 25 μM DCFDA diluted in serum-free adhesion medium without phenol red, according to the manufacturer’s instructions (ab113851, DCFDA Cellular ROS Detection Assay Kit, Abcam, Toronto, ON, Canada). End-point fluorescence from triplicate wells for each experimental condition was measured using a fluorescence microplate reader (1420 Multilabel Counter, Perkin Elmer, Waltham, MA, USA) with settings of 485 nm excitation and 535 nm emission.

### 4.8. Real-Time PCR Analysis

Total RNA was isolated from cells using Trizol reagent (ThermoFisher Scientific, Waltham, MA, USA). One microgram of total mRNA was reverse transcribed into complementary DNA (cDNA) using the QuantiTect reverse transcription kit (Qiagen, Montreal, QC, Canada) according to the manufacturer’s protocol. Quantitative real-time PCR (qPCR) was performed using SYBR Select Master Mix (Applied Biosystems, Foster City, CA, USA) to quantify the transcription levels of genes implicated in de novo lipogenesis and endoplasmic reticulum stress (ER stress). Primer sequences are presented in Table S2. Results are presented as arbitrary units indicating relative expression based on the comparative Ct (ΔΔCt) method. Data were normalized using the housekeeping β-Actin gene and expressed as fold changes relative to control samples for ER stress genes.

### 4.9. Analysis of Cell Death by Flow Cytometry

Cells were seeded in 6-well plates and treated 24 h after seeding as indicated above, and then harvested 6 days after. For cell death analysis, all the cells were incubated for 30 min at room temperature (RT) with BV421 AnnexinV (563973, BD Biosciences, Mississauga, ON, Canada) (dilution 1/10) and 5 min at RT with 0.9 nM of DRAQ7 (ab109202, Abcam Inc.). In addition, 10,000 events were counted per condition using the Fortessa flow cytometer (BD Biosciences) and analyzed with FlowJo software, version10.6.

### 4.10. Murine Xenograft Model

All animal experiments complied with relevant ethical regulations for animal testing and research at CRCHUM. They were done with approval from our institutional committee on animal care (CIPA) under the protocol number C18016AMMs. LNCaP and C4-2B cells in exponential growth phase were prepared respectively at a concentration of 1 million cells and 5 million cells in 200 μL of PBS-matrigel (*v*/*v*). NRG mice (NOD-Rag1null IL2rgnull, NOD rag gamma) were obtained from the Jackson Laboratory (The Jackson Laboratory, Bar Harbor, ME, USA). All experiments were carried out with male mice with an average age of 12-weeks. To initiate tumor xenografts, 0.2 mL of cell suspension were injected into the left flank. Mice were weighed, and tumor volumes were measured twice per week. When tumor volume reached 300–400 mm^3^, mice were randomized into 4 groups of 6–10 mice: untreated, treated with ENZ, treated with SCD1 INH, or treated with combination. ENZ (20 mg/kg) was injected intraperitoneally (100 μL per injection) daily for 3 weeks and SCD1 INH (30 mg/kg) was initially administered orally in Nutrigel diet (Product#S5769, Bio-Serv, Flemington, NJ, USA) for one week then by gavage (100 μL per gavage) every day for the subsequent 2 weeks.

### 4.11. Tumor Lipid Content

Tumors were processed for quantitative profiling of fatty acid content by gas chromatography-mass spectrometry using previously described methods [54,55,56]. Briefly, pulverized tissues (50 mg) were incubated overnight at 4 °C in a solution of chloroform/methanol (2:1) containing 0.004% butylated hydroxytoluene (BHT), filtered through gauze and dried using nitrogen gas. Lipids were eluted on an aminopropyl column (Bond Elut LRC-NH2, 500 mg) (Agilent Technologies Inc., Santa Clara, CA, USA). Total fatty acids were analyzed as their methyl esters after direct transesterification with acetyl chloride/methanol on a 7890B gas chromatograph coupled to a 5977A mass selective detector (Agilent Technologies Inc., Santa Clara, CA, USA) equipped with a capillary column (J&W Select FAME CP7420; 100 m × 250 µm inner diameter; Agilent Techonologies Inc., Santa Clara, CA, USA) and operated in the PCI mode using ammonia as reagent gas. Samples were analyzed under the following conditions: injection (3 µL) at 270 °C in a split mode (split ratio 50:1) using high-purity helium as the carrier gas (constant flow rate: 0.44 mL/min) and the following temperature gradient: 190 °C for 25 min, increased by 1.5 °C/min until 236 °C. Fatty acids were analyzed as their [M + NH_3_]+ ions by selective ion monitoring and concentrations were calculated using standard curves and isotope-labeled internal standards.

### 4.12. Statistical Analysis

Data are expressed as means ± SD. To compare values obtained from three or more groups, one-way ANOVA was performed. A *p*-value of <0.05 was considered significant.

## 5. Conclusions

In summary, we have demonstrated that de novo lipogenesis is highly enhanced in CRPC and ENZR cells compared to HSPC. Interestingly, we showed that inhibition of de novo lipogenesis via the inhibition of SCD1 induced cell death of PC cells. This observation led to the use of combination therapy of SCD1 inhibitor and Enzalutamide for PC in order to improve response, delay enzalutamide resistance and improve disease free progression. We showed that the combination therapy affects the lipid content in tumor cells by inducing a decrease in MUFA that affects the activation of PI3K/AKT pathway, a major oncogenic pathway in PC, resulting in diminished cell proliferation. This effect is further compounded by an increase in SFA content inducing ER stress and a high level of ROS content, resulting in greater cell death.

This strategy provides a strong rationale to combine an AR targeted therapy such as ENZ with an inhibitor of SCD1 for the treatment of PC patients with advanced disease.

## Supplementary Materials

The following are available online at https://www.mdpi.com/2072-6694/12/11/3339/s1, Table S1: List of primary antibodies, Table S2: List of primers, Figure S1: Inhibition of SCD1 using siRNA in combination with ENZ affects significantly the proliferation of PC cells, Figure S2: The whole blots.
