# Supplementary material for: Modulation of de Novo Lipogenesis Improves Response to Enzalutamide Treatment in Prostate Cancer
Source: Cancers (Basel). 2020 Nov 11;12(11):3339. doi: 10.3390/cancers12113339 (PMC7698241; doi:10.3390/cancers12113339)
Supplement: Supplementary file 1 [file cancers-12-03339-s001.pdf]

*Supplementary Materials:*

# Modulation of de novo Lipogenesis Improves Response to Enzalutamide Treatment in Prostate Cancer

**Mohamed Amine Lounis, Benjamin Péant, Kim Leclerc-Desaulniers, Dwaipayan Ganguli, Caroline Daneault, Matthieu Ruiz, Amina Zoubeidi, Anne-Marie Mes-Masson and Fred Saad**

**Table S1.** List of primary antibodies.

| Name | Cat Number | Isotype | Company | Dilution |
|---|---|---|---|---|
| SCD1 | Ab39969 | Rabbit IgG | Abcam | 1:1000 |
| PERK | #5683 | Rabbit IgG | Cell signaling | 1:1000 |
| CHOP | #2895 | Mouse IgG | Cell signaling | 1:1000 |
| IREα | #3294 | Rabbit IgG | Cell signaling | 1:1000 |
| Cleaved PARP | #5625 | Rabbit IgG | Cell signaling | 1:1000 |
| pPDK1 (ser241) | #3438 | Rabbit IgG | Cell signaling | 1:1000 |
| PDK1 (D4Q4D) | #13037 | Rabbit IgG | Cell signaling | 1:1000 |
| pAKT (ser473) | #4060 | Rabbit IgG | Cell signaling | 1:1000 |
| AKT | #3685 | Rabbit IgG | Cell signaling | 1:1000 |
| β-ACTIN | ab8226 | Mouse IgG | Abcam | 1:5000 |

**Table S2.** List of primers.

| Name | Forward Sequence | Reverse Sequence |
|---|---|---|
| SREBF1 | 5′-GCCCCTGTAACGACCACTG-3′ | 5′-CAGCGAGTCTGCCTTGATG-3′ |
| ACC | 5′- ATGTCTGGCTTGCACCTAGTA-3′ | 5′-CCCCAAAGCGAGTAACAAATTCT-3′ |
| FASN | 5′-AAGGACCTGTCTAGGTTTGATGC-3′ | 5′-TGGCTTCATAGGTGACTTCCA-3′ |
| SCD | 5′-TCTAGCTCCTATACCACCACCA-3′ | 5′-TCGTCTCCAACTTATCTCCTCC-3′ |
| ELVOL6 | 5′-AACGAGCAAAGTTTGAACTGAGG-3′ | 5′-TCGAAGAGCACCGAATATACTGA-3′ |
| ATF4 | 5′-ATGACCGAAATGAGCTTCCTG-3′ | 5′-GCTGGAGAACCCATGAGG-3′ |
| ATF3 | 5′-CCTCTGCGCTGGAATCAGTC-3′ | 5′-TTCTTTCTCGTCGCCTCTTTTT-3′ |
| GRP78 | 5′-CATCACGCCCTCCTATGTCG-3′ | 5′-CGTCAAAGACCGTGTTCTCG-3′ |
| XBP1 | 5′-CCCTCCAGAACATCTCCCCAT-3′ | 5′-ACATGACTGGGTCCAAGTTGT-3′ |
| CHOP | 5′-GGAAACAGAGTGGTCATTCCC-3′ | 5′-CTGCTTGAGCCGTTCATTCTC-3′ |
| β-Actin | 5′- CATGTACGTTGCTATCCAGGC-3′ | 5′-CTCCTTAATGTCACGCACGAT-3′ |

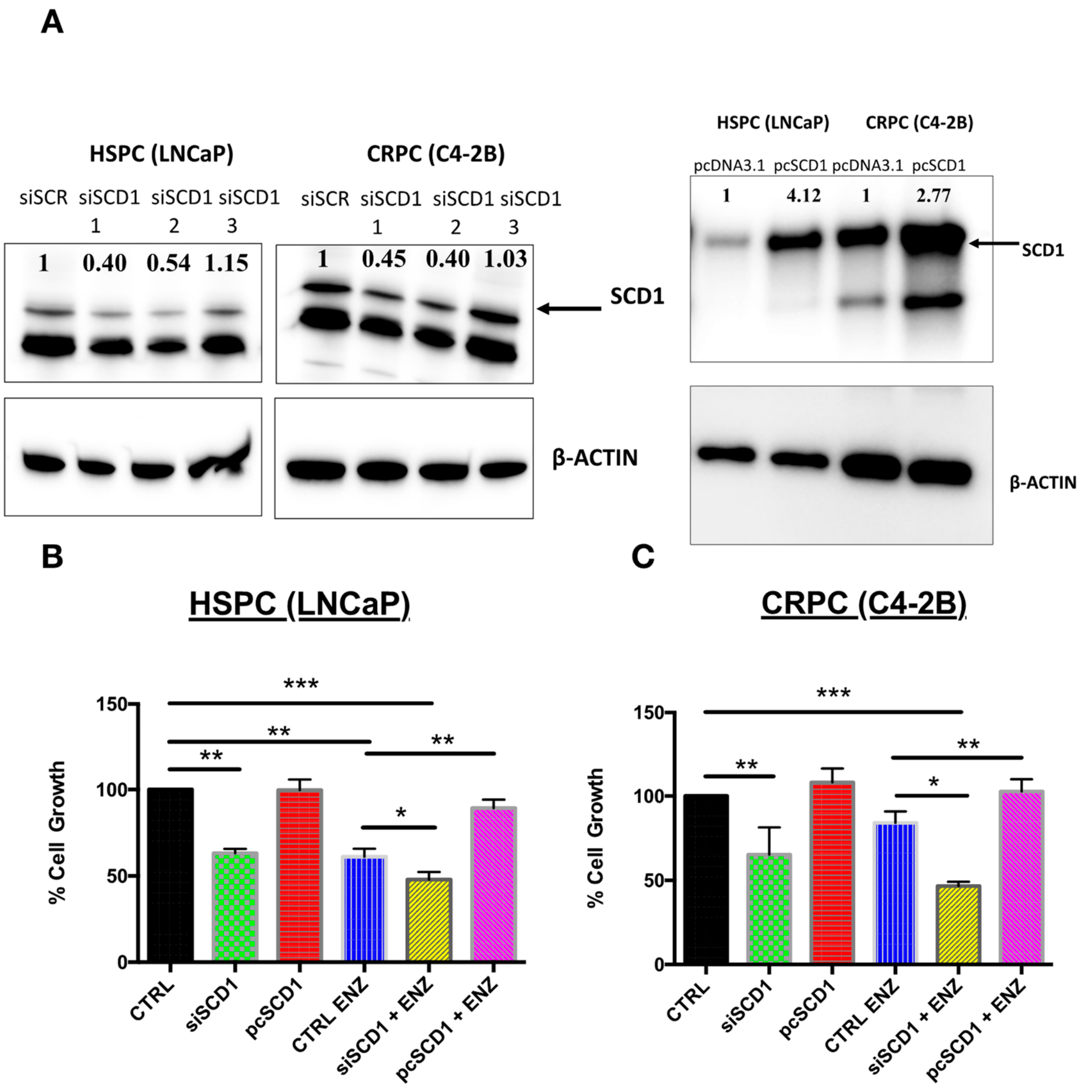


**Figure S1.** Inhibition of SCD1 using siRNA in combination with ENZ affects significantly the proliferation of PC cells. (**A**) Western blot analyses of SCD1 protein from HSPC (LNCaP) and CRPC (C4-2B) cells transfected with SCD1 siRNAs (siSCD1), siSramble, (Left panel) and with pcDNA SCD1 (pcSCD1) or pcDNA vector (Right panel). Proliferation of LNCaP (**B**) and C4-2B (**C**) cells transfected with SCD1 (siSCD1), siSramble, pcDNA SCD1 (pcSCD1) or pcDNA vector in absence or in presence of 10 μM ENZ after 3 days of treatment. Data represent the mean ± SD of three independent experiments. Data were analyzed using the one-way ANOVA test. * $p < 0.05$, ** $p < 0.01$, and *** $p < 0.001$.

**Figure 2D**

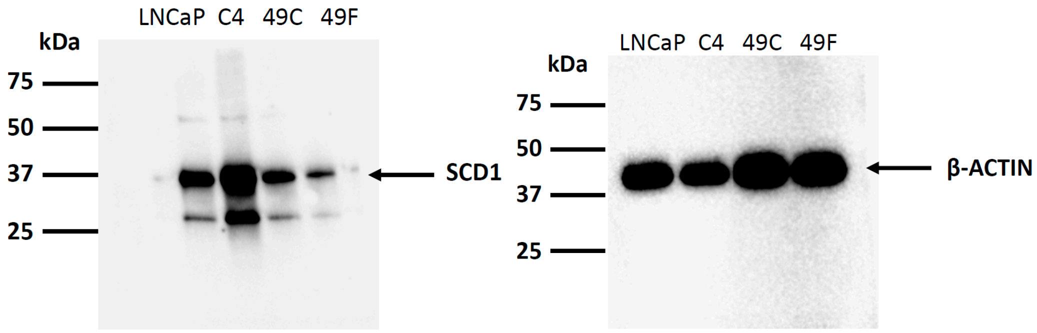


# Figure 3D

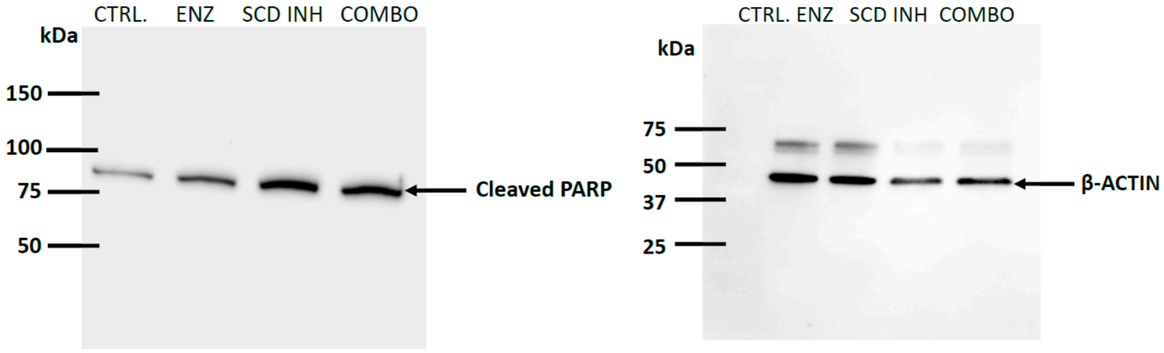


# Figure 3E

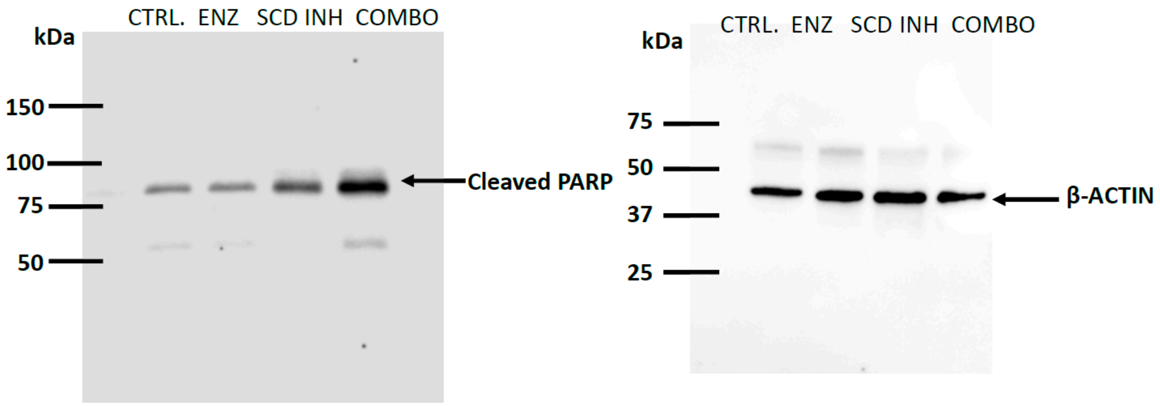

# Figure S1

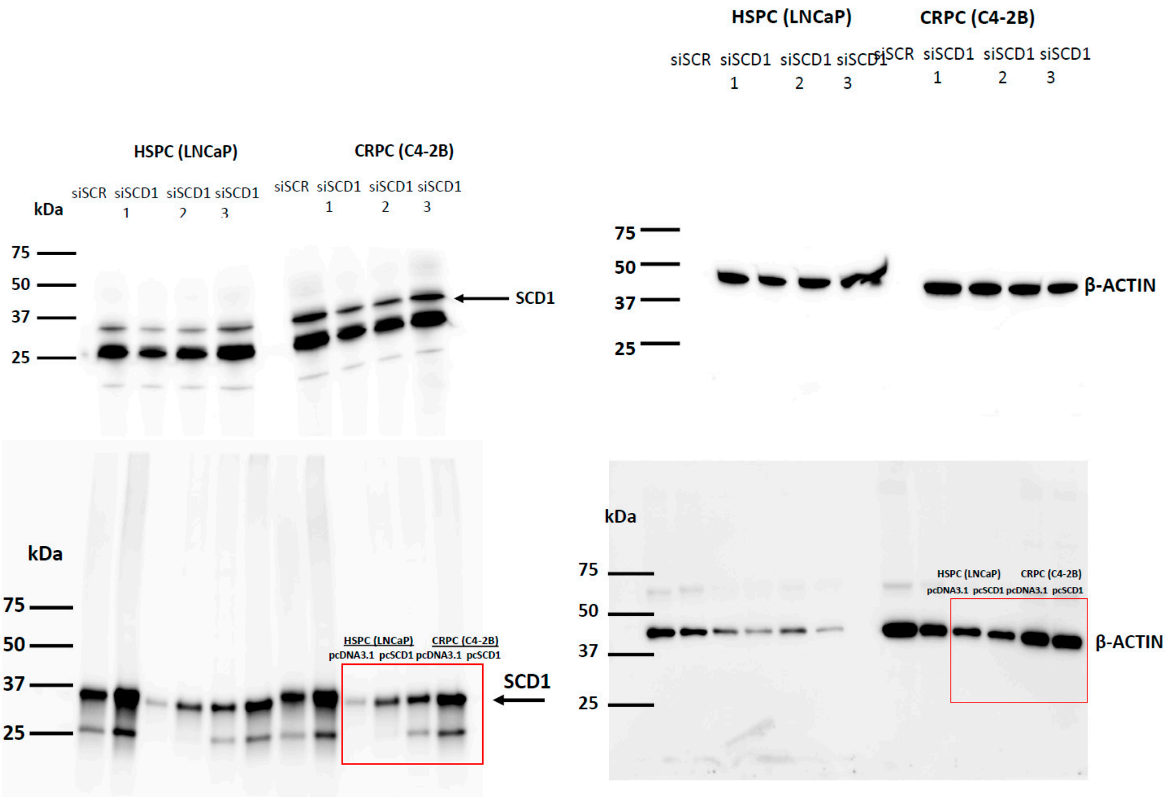


# Figure 4A

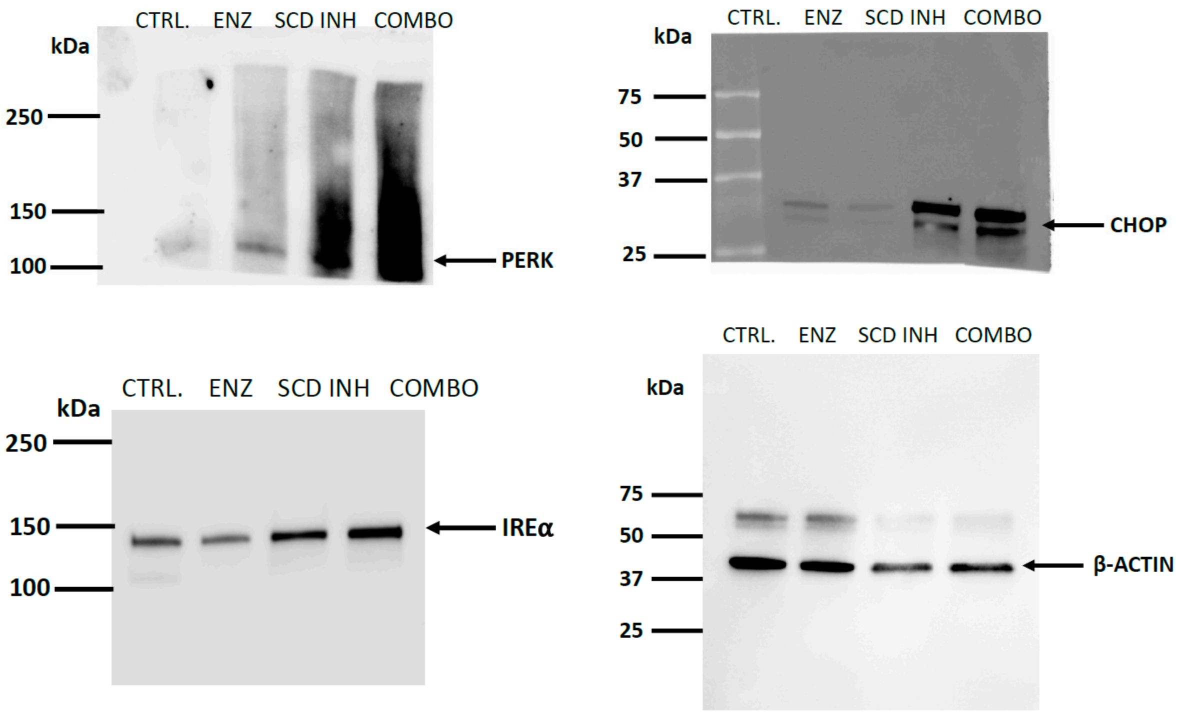

**Figure 4B**

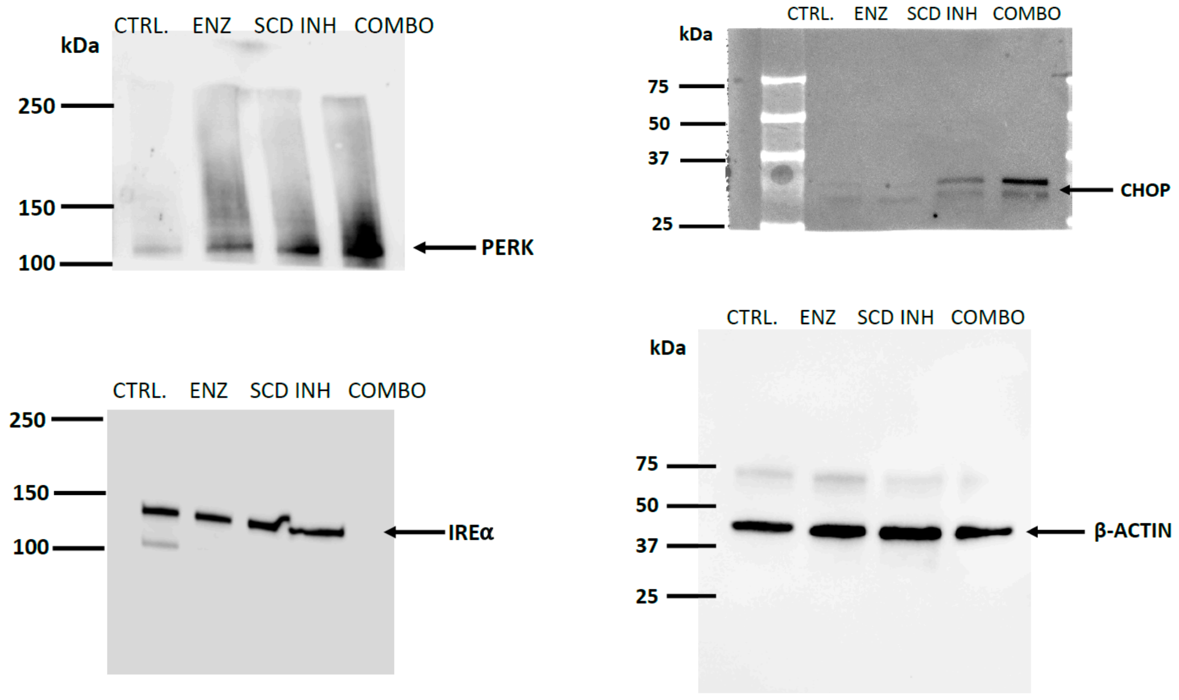


**Figure 5A-B**

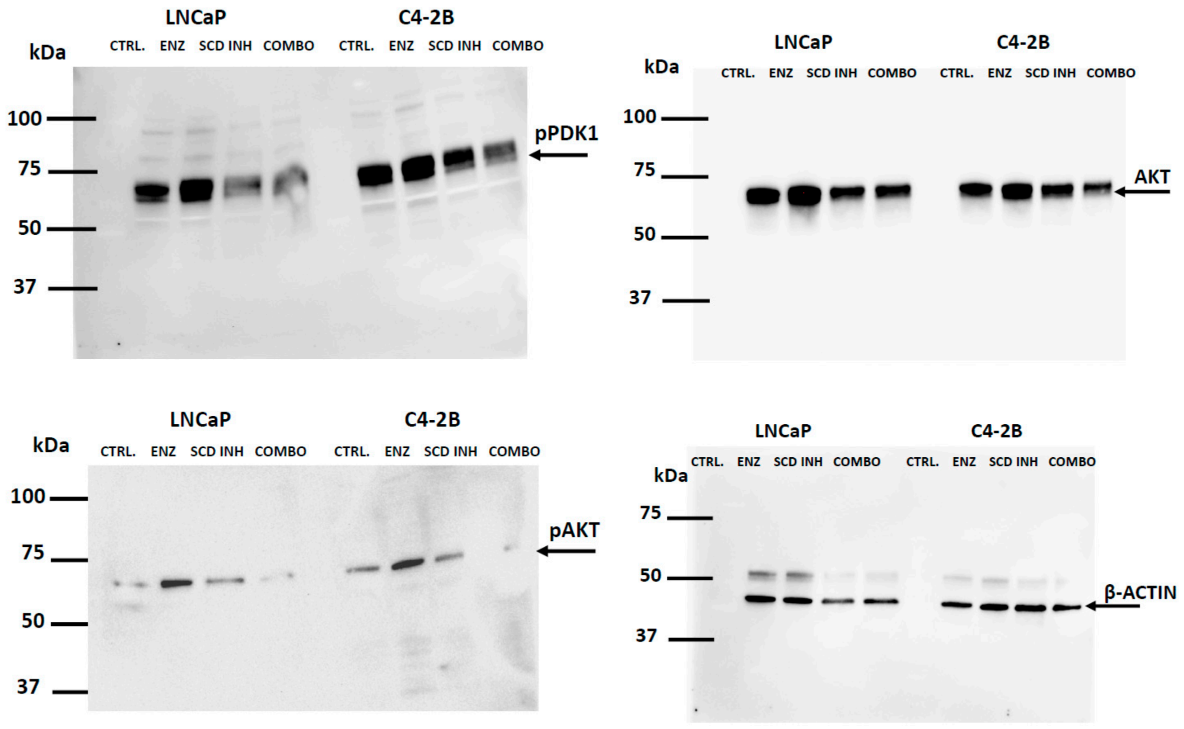

## Figure 5C

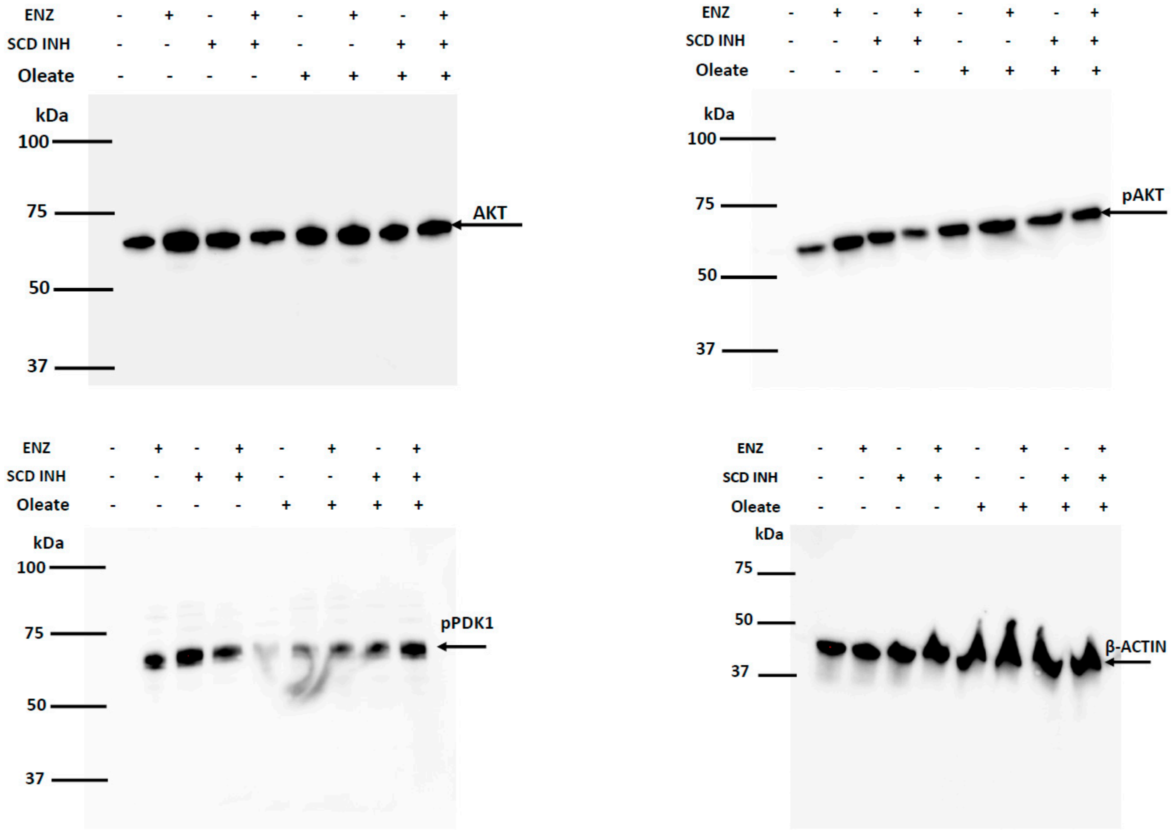


## Figure 5D

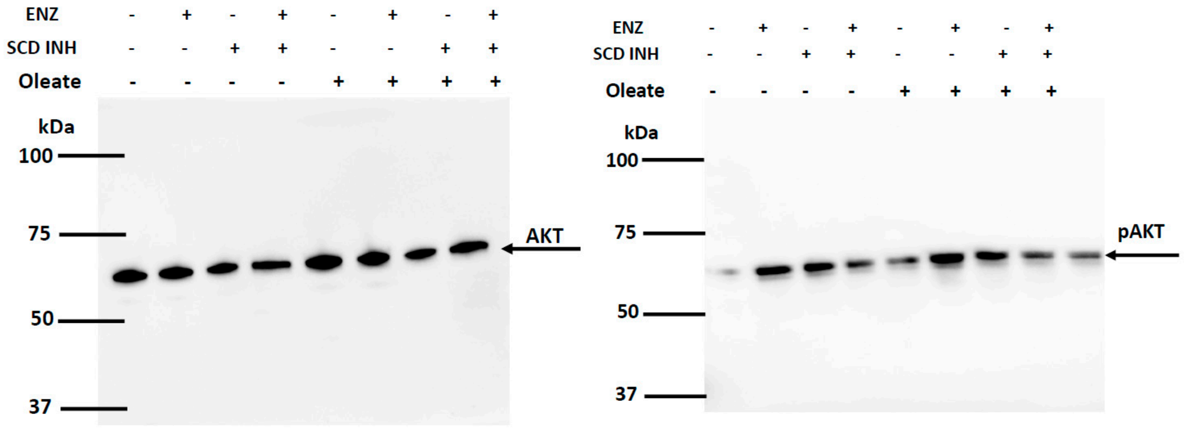

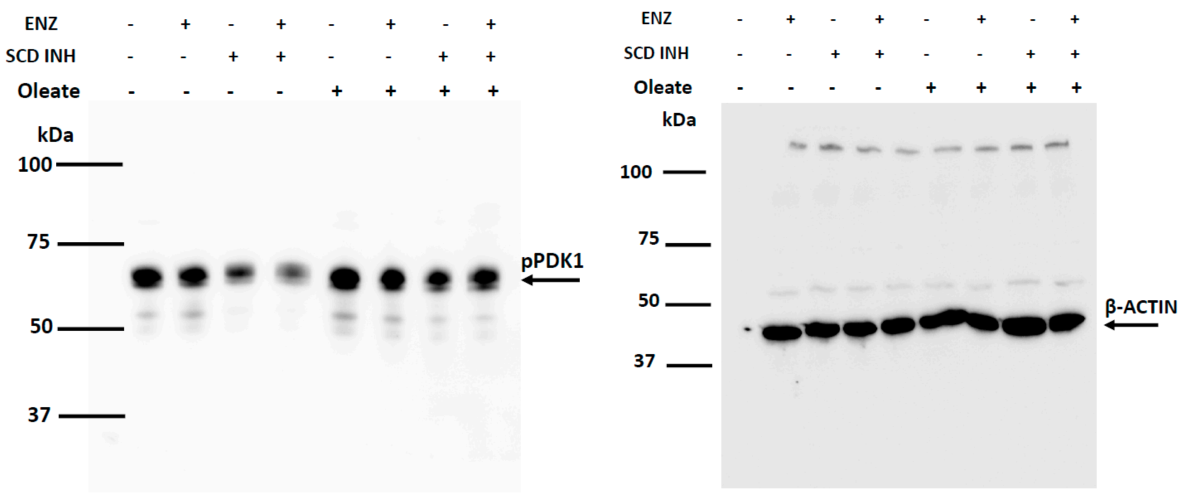


**Figure 6C**

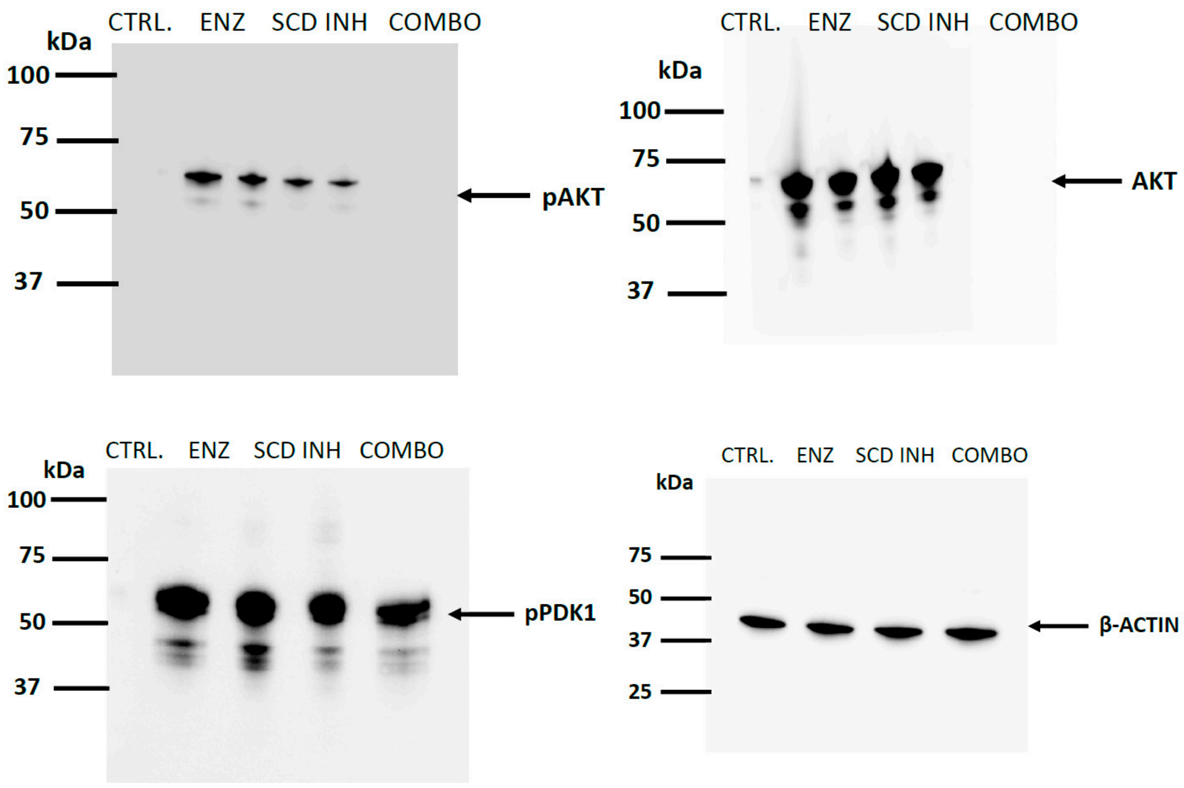


**Figure S2.** The whole blots.

© 2020 by the authors. Licensee MDPI, Basel, Switzerland. This article is an open access article distributed under the terms and conditions of the Creative Commons Attribution (CC BY) license (http://creativecommons.org/licenses/by/4.0/).
